# Oral appliance therapy vs. positional therapy for managing positional obstructive sleep apnea; a systematic review and meta-analysis of randomized control trials

**DOI:** 10.1186/s12903-024-04277-8

**Published:** 2024-06-07

**Authors:** Abdelrahman MA Mohamed, Omar Magdy Mohammed, Shanshan Liu, Maher Al-balaa, Leena Ali Al-warafi, Song Juan Peng, Yi Qiang Qiao

**Affiliations:** 1 Department of Orthodontics, 1st Affiliated Hospital, Zhengzhou University, Zhengzhou, China; 2 Member (MOrth RCSEd), Royal College of Surgeon, Edinburgh, UK; 3 Department of General Surgery, Faculty of Medicine, Fayoum University, Fayoum, Egypt; 4 Private practice, Zhongshan squirrel dental hospital, Guangdong, China

**Keywords:** Obstructive sleep apnea, Oral appliance therapy, Positional therapy, Sleep position trainer, Mandibular advancement device therapy

## Abstract

**Aim:**

To assess the efficacy of positional therapy and oral appliance therapy for the management of positional obstructive sleep apnea.

**Methods:**

We searched PubMed, Web of Science, Cochrane, and SCOPUS for relevant clinical trials. Quality assessment of the included trials was evaluated according to Cochrane’s risk of bias tool. We included the following outcomes: The apnea-hypopnea index (AHI), AHI non-supine, AHI supine, sleep efficiency, percentage of supine sleep, Adherence (≥ 4 h/night, ≥ 5 days/week), Oxygen desaturation Index, Arousal Index, Epworth Sleepiness Scale score (ESS), Mean SpO2, and Functional Outcomes of Sleep Questionnaire.

**Results:**

The AHI non-supine and the ESS scores were significantly lower in the OAT cohort than in the PT cohort. The PT cohort was associated with a significantly decreased percentage of supine sleep than the OAT cohort (MD= -26.07 [-33.15, -19.00], *P* = 0.0001). There was no significant variation between PT cohort and OAT cohort regarding total AHI, AHI supine, ODI, sleep efficiency, arousal index, FOSQ, adherence, and mean SpO2.

**Conclusion:**

Both Positional Therapy and Oral Appliance Therapy effectively addressed Obstructive Sleep Apnea. However, Oral Appliance Therapy exhibited higher efficiency, leading to increased supine sleep percentage and more significant reductions in the Apnea Hypopnea Index during non-supine positions, as well as lower scores on the Epworth Sleepiness Scale.

**Supplementary Information:**

The online version contains supplementary material available at 10.1186/s12903-024-04277-8.

## Introduction

Obstructive sleep apnea (OSA) is characterized by episodes of the airway collapsing completely or partially, arousing the patient from sleep or causing a drop in oxygen saturation [1]. Fragmented, non-restorative sleep is the result of this disturbance. Positional Obstructive Sleep Apnea (POSA) has major effects on mental illness, cardiovascular health, driving safety, and quality of life [2]. A number of mechanisms are probably involved in the pathophysiology of pharyngeal narrowing and closing during sleep, which is a complex event [3]. Posterior airway dimensions which are affected by age, obesity, race, or genetic reasons may predispose to breathing disorders during sleep [4, 5]. Upper airway blockage during sleep is likely caused by diminished ventilatory drive associated with sleep, as well as anatomic and neuromuscular risk factors [6].

An in-laboratory polysomnogram is the gold standard for the diagnosis of POSA. The two most prevalent signs of POSA are daytime sleepiness and snoring [7]. Others include bed partner-reported apneas, gasping or choking when you wake up, frequent awakenings, restless sleep, erectile dysfunction, and nocturia. Patients with POSA may have restless sleep, headaches in the morning, low energy, bad mood, fatigue, or poor concentration [8]. There are different options for POSA treatment, such as behavioral treatments, including positional therapy, weight loss, and avoidance of sedatives and/or alcohol before bedtime [9]. The avoidance of sleeping in a supine position to counteract the gravitational impact of supine sleep on the tongue and airway narrowing is known as positional therapy (PT) [10]. There are several methods for attempting to remain off the back when sleeping. Tennis balls can be placed in a tube sock and attached vertically in the center of the back of a sleep shirt, or a loaded backpack can be worn to bed. There are also FDA-approved commercially accessible positional therapy devices [11, 12].

Continuous positive airway pressure (CPAP) is still regarded as the gold standard of care more than three decades after it was first used [13, 14]. Nasal CPAP (nCPAP) is very effective at treating sleep apnea-related clinical sequelae and controlling symptoms while also enhancing quality of life [15]. There are other positive airway pressure treatment options for individuals who cannot tolerate CPAP or require high amounts of positive pressure [16]. Oral appliance therapy (OAT) and mandibular advancement devices (MAD), especially if they are custom-made, are efficient in treating mild to severe POSA and offer a good substitute for patients who are unable to tolerate CPAP therapy [17, 18]. Polysomnography POSA indicators are improved with OAT. In a sleep study, oxygen saturation, respiratory event indices, sleep efficiency, and arousal index (AI) all showed improvements from their baseline values [19].

Our systematic review and meta-analysis aimed to compare the efficacy of PT with that of OAT for the management of POSA. The null hypothesis posits that there is no difference between Positional Therapy (PT) and Oral Appliance Therapy (OAT) in the management of Positional Obstructive Sleep Apnea (POSA).

## Methods

We conducted our study based on the PRISMA recommendations and guidelines [20] and registered our study on PROSPERO (CRD42024517491).

### Search strategy and information sources

Two authors developed a search strategy by combining these keywords: (“Obstructive sleep apnea” OR “sleep apnea” OR “positional sleep apnea” OR “POSA”) AND (“mandibular advancement” OR “oral appliance” OR “oral appliance devices” OR “OAT”) AND (“positional therapy” OR “sleep position trainer”). Concerning data sources, we searched SCOPUS, PubMed, Cochrane Library, and Web of Science databases in the search process till December 2023 for articles that matched our inclusion criteria.

### Study selection

In three stages, Two authors screened the studies that were included. The first stage required using EndNote Software to import the results from electronic databases into a Microsoft Excel sheet [22]. The articles that were imported into the Excel sheet were screened for titles and abstracts as part of the second stage. The third stage was the full-text screening of the step 2 citations that were included. Furthermore, we conducted a manual review of the references of the included publications to identify any potential undiscovered research. We selected the eligible articles according to the following eligibility criteria:

- **Population**: Adult individuals suffering from POSA.
- **Intervention**: Patients underwent PT.
- **Comparator**: Patients underwent OAT.
- **Outcomes**. The apnea-hypopnea index (AHI), AHI non-supine, AHI supine, sleep efficiency, percentage of supine sleep, Adherence (≥ 4 h/night, ≥ 5 days/week), Oxygen desaturation Index (ODI), Arousal Index (AI), Epworth Sleepiness Scale score (ESS), Mean SpO2 (peripheral capillary oxygen saturation), and Functional Outcomes of Sleep Questionnaire (FOSQ).
- **Study design**: we included only randomized clinical trials (RCTs) and excluded meta-analyses, observational studies, surveys, abstracts, and reviews.

### Inclusion and exclusion criteria

Inclusion criteria for the mentioned objectives included research completed in the adult age group (above 18 years old). We included studies that were published in the English language from the year 2000 to January 2024. We only included RCTs that involved adult patients with POSA and included two comparators (PT vs. OAT). We excluded articles not in English, published before the year 2000, did not have our main outcomes, were meta-analyses, observational studies, surveys, abstracts, reviews, and single-arm RCTs that had no comparators (no control group).

### Quality assessment

Since we involved only RCTs, we utilized the Cochrane risk of bias tool, which depends on assessing eight domains in each clinical trial [21]. Each domain could be categorized as high, unclear, or low risk of bias.

### Data extraction

We extracted three types of data from the involved articles: the first category is the demographic characteristics of the involved patients and the baseline values of our outcomes. The second category was extracting data of the following outcomes for analysis: The apnea-hypopnea index (AHI), AHI non-supine, AHI supine, sleep efficiency, percentage of supine sleep, Adherence (≥ 4 h/night, ≥ 5 days/week), Oxygen desaturation Index (ODI), Arousal Index (AI), Epworth Sleepiness Scale score (ESS), Mean SpO2, and Functional Outcomes of Sleep Questionnaire (FOSQ). The last category was data of quality assessment. The process of data collection was conducted using Microsoft Excel [22]. Three of the authors had roles in collecting data and data extraction. Each one of them extracted the three categories, and after they finished, another author revise the extracted data of each one and compared them to find any mistakes.

### Statistical analysis

We performed this meta-analysis using Review Manager Software [23]. Our study involved continuous outcomes. We used a 95% confidence interval (CI) and mean difference (MD) to analyze continuous data. When data were homogenous, the fixed-effects model was employed; when data were heterogeneous, the random-effects model was utilized. We used the I^2^ and p-value of the Chi-square tests to assess the degree of consistency between the studies [24]. Values of *P* < 0.1 or I^2^ > 50% were significant indicators of the presence of heterogeneity.

## Results

### Summary of the included studies

**Fig. 1:**
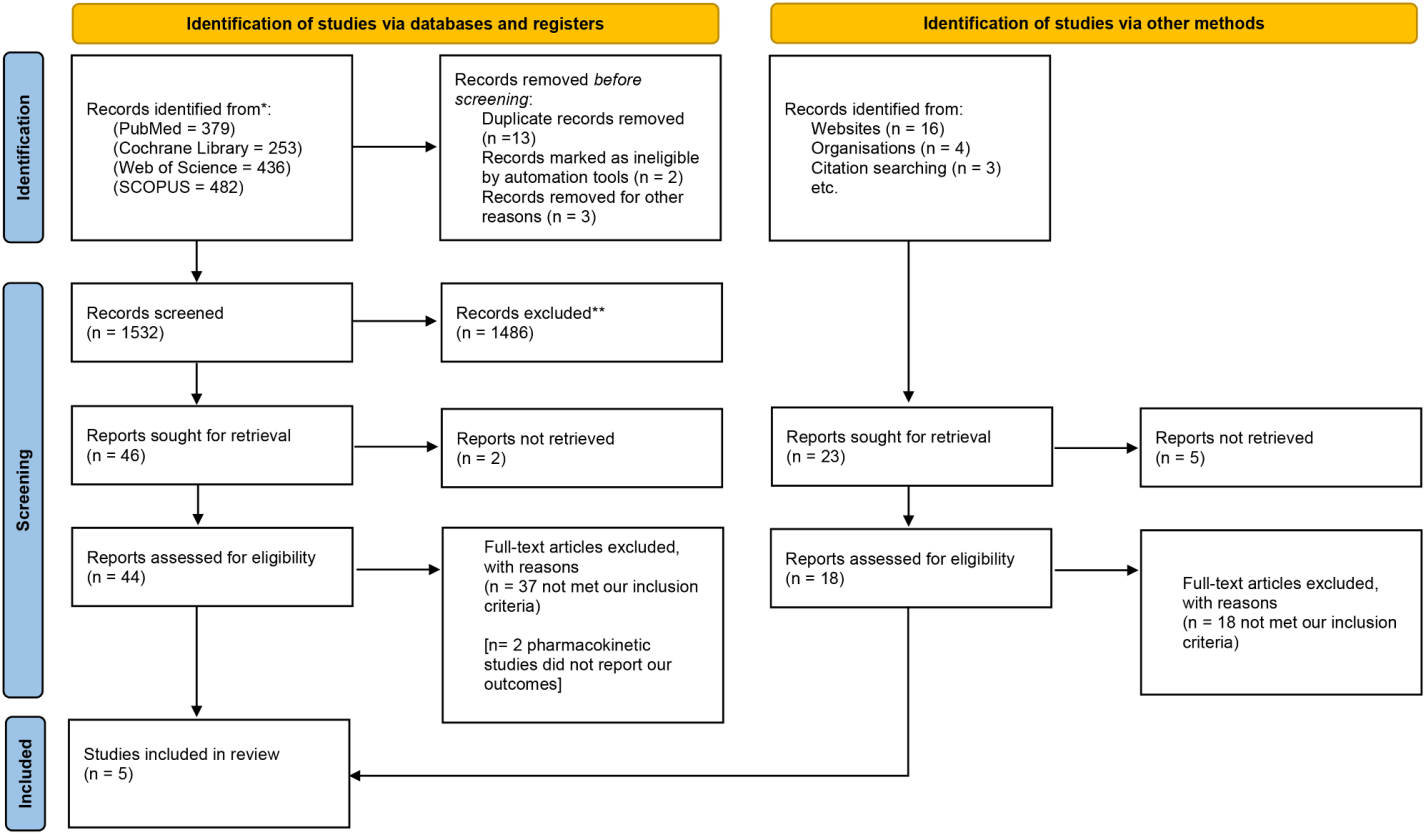
Shows a PRISMA flow diagram of our literature search

The literature search results are illustrated in the PRISMA flow diagram in Fig. 1. Our study involved five RCTs [25–29], which included a total of 377 patients suffering from positional POSA. The PT cohort included 130 males and 57 females, while the OAT cohort included 137 males and 53 females. The mean age of participants in the PT cohort was 46 years old, while the OAT cohort was 45.5 years old. Most of the trials we included are recent trials that were performed in different countries (China, Netherlands, Japan, and Belgium). The follow-up duration was three months in all the included studies except in Huang et al., which was six months. Tables 1 and 2 demonstrate the demographics and baseline characteristics of the involved patients and RCTs.

**Table 1:** Demonstrates the demographics and baseline characteristics of the involved patients and RCTs

Study ID		Benoist 2017	De Ruiter 2018	Dieltjens 2015
Location		Netherlands	Netherlands	Belgium
Duration		3 months	3 months	3 months
Sample size, n	PT	48	29	20
	OAT	51	29	
Age(years), mean	PT	47.3 ± 10.1	49.5 ± 9.4	52.5 ± 10.5
	OAT	49.2 ± 10.2	43.8 ± 10.3	
BMI, kg/m2	PT	27.5 ± 2.9	27.7 ± 2.8	26.4 ± 3.0
	OAT	27.7 ± 4.5	27.1 ± 2.9	
Male, (%)	PT	34 (70.8)	19 (65.5)	12 (58)
	OAT	36 (70.6)	15 (51.7)	
female, (%)	PT	14 (29.2)	10 (34.5)	8 (42)
	OAT	15 (29.4)	14 (48.3)	
Neck circumference, cm	PT	38.0 ± 3.6	37.9 ± 3.8	NR
	OAT	37.7 ± 3.2	38.3 ± 3.4	
Smoking, n (%)	PT	11 (22.9)	5 (17.2)	NR
	OAT	12 (23.5)	6 (31.6)	
Alcohol intake, n(%) ≤ 2drinks/day	PT	45 (93.7)	26 (89.7)	NR
	OAT	48 (94.1)	19 (65.5)	
AHI, events/hour	PT	13.0 [9.7–18.5]	13.2(10.2–19)	20.9 (17–34)
	OAT	11.7 [9.0-16.2]	12.1 (7–17.2)	
AHI supine, events/hour	PT	27.0 [18.7–43.1]	28.5 (18.9–46.2)	39.1 (26.4; 58.2)
	OAT	25.8 [17.4–35.0]	26 (11.6–36.8)	
Percentage supine sleep	PT	44.5 [30.0-55.5]	41 (30–54)	20.9 (17–34)
	OAT	39.0 [26.0–54.0]	47 (25.0–57)	
non-supine AHI, events/hour	PT	NR	4.1 (2.4–5.8)	11.1 (6.3; 26.1)
	OAT		2.4 (0.9–5.7)	
ODI, events/h	PT	NR	9 (7–15.5)	7.7 (6.6; 16.5)
	OAT		13 (7–16)	
Sleep efficiency	PT	NR	92 (84–95.5)	
	OAT		92 (89–94)	

Values are mean ± standard deviation, median (interquartile range), or number of patients (%). AHI apnea hypopnea index, OAT oral appliance therapy, ODI oxygen desaturation index, PT Positional therapy

**Table 2:** Demonstrates the demographics and baseline characteristics of the involved patients and RCTs

Study ID		Huang 2023	Suzuki 2021
Location		China	Japan
Duration		6 months	3 months
Sample size, n	PT	20	80
	OAT	20	80
Age(years), mean	PT	39.20 ± 10.92	45.6 ± 11.4
	OAT	41.55 ± 11.79	47.5 ± 11.4
BMI, kg/m2	PT	23.91(22.97–25.75)	25.2 ± 3.8
	OAT	25.19 (23.53–26.78)	24.9 ± 3.2
Male, (%)	PT	17 (85)	54 (67.5)
	OAT	18 (90)	62 (77.5)
female, (%)	PT	3 (15)	26 (32.5)
	OAT	2 (10)	18 (22.5)
Neck circumference, cm	PT	NR	NR
	OAT		
Smoking, n (%)	PT	5 (25)	NR
	OAT	6 (30)	
Alcohol intake, n(%) ≤ 2drinks/day	PT	2 (10)	NR
	OAT	2 (10)	
AHI, events/hour	PT	19.21 (11.77–23.9)	24.2 ± 17.1
	OAT	18.58 (16.1-24.55)	20.8 ± 11.2
AHI supine, events/hour	PT	24.4 (18.13–39.05)	37.4 ± 19.0
	OAT	27.4 (21.8-36.93)	31.6 ± 16.6
Percentage supine sleep	PT	62.98 (42.31–83.11)	NR
	OAT	64.88 (49.48–73.36)	
non-supine AHI, events/hour	PT	4.72 (1.54–8.75)	13.2 ± 12.1
	OAT	4.82 (1.14–8.67)	9.4 ± 9.1
ODI, events/h	PT	17.5 (9.6-23.13)	NR
	OAT	15.85 (12.63–21.6)	
Sleep efficiency	PT	75.77 ± 13.42	78.6 ± 16.4
	OAT	75.51 ± 11.53	77.5 ± 11.2

Values are mean ± standard deviation, median (interquartile range), or number of patients (%). AHI apnea hypopnea index, OAT oral appliance therapy, ODI oxygen desaturation index, PT Positional therapy

### Results of the quality assessment

**Fig. 2:**
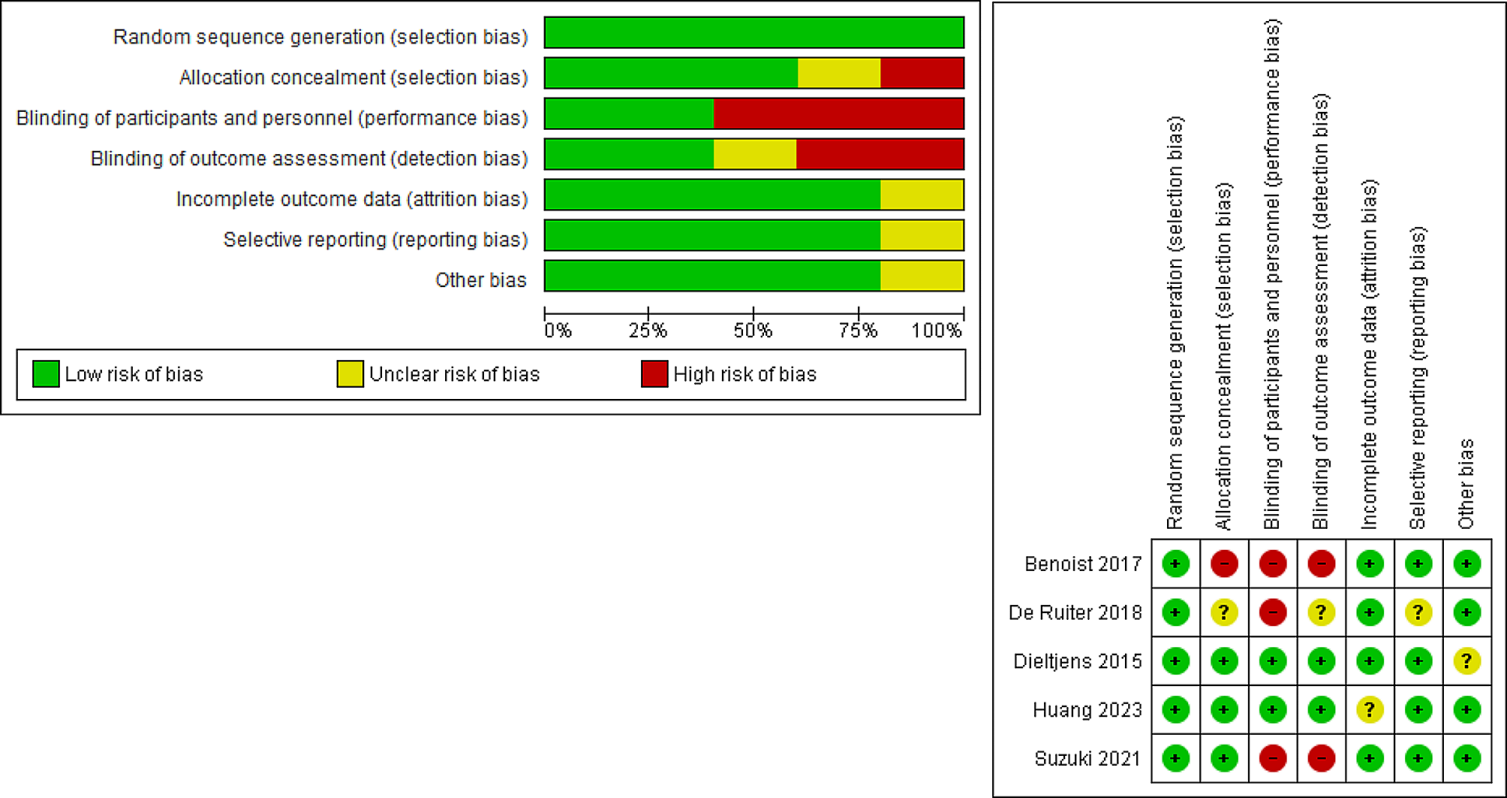
Summary of the risk of bias of included studies

After using Cochrane’s risk of bias tool for the evaluation of the included RCTs, we found that all the included RCTs were randomized, while three of them [26–28] were at low risk of allocation concealment. Additionally, two trials [26, 27] were at low risk of blinding participants, personnel, and outcome assessment. The overall assessment of the Risk of Bias (ROB) revealed that the included RCTs were at low ROB. Figure 2 shows the ROB assessment of the involved RCTs.

### Analysis of the outcomes

#### Total AHI, events/hour

**Fig. 3:**
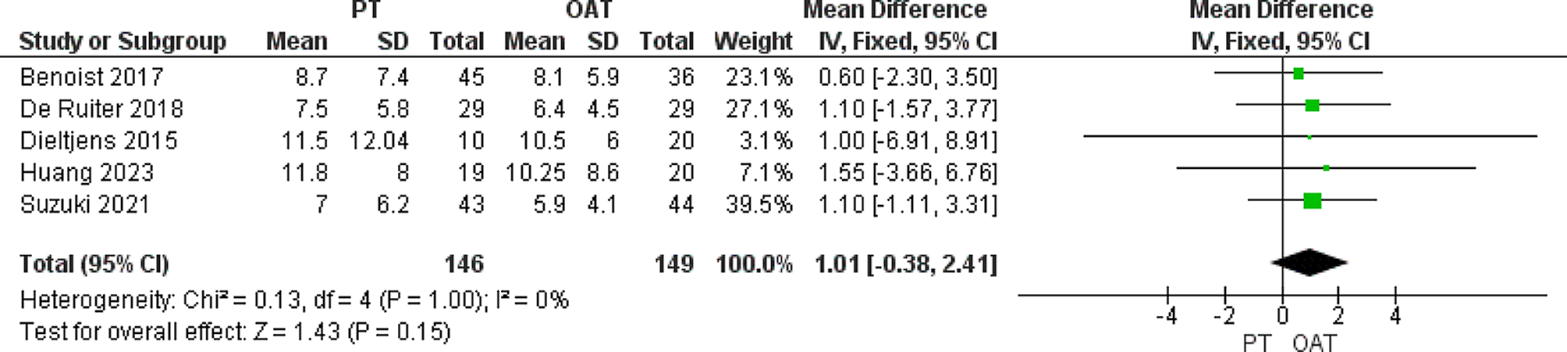
Heterogeneity and overall effect of total AHI that does not favor any of both groups

All the included studies reported the total AHI. Our analysis proved that there was a similarity between both cohorts (MD = 1.01 [-0.38, 2.41], *P* = 0.15). The pooled data showed homogeneity (*P* = 0.1, I^2^ = 0%) (Fig. 3).

#### AHI supine, events/hour

**Fig. 4:**
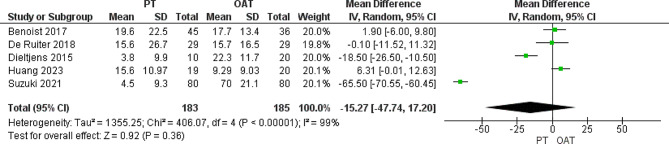
Heterogeneity and the overall effect of AHI supine that does not favor any of both groups

AHI supine was reported by all the included trials. We found that there was no substantial difference between both cohorts (MD= −15.27 [−47.74, 17.20], *P* = 0.36). The pooled data showed homogeneity (*P* = 0.0001, I^2^ = 99%) (Fig. 4).

#### AHI non-supine, events/hour

**Fig. 5:**
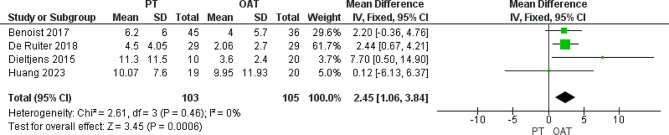
Heterogeneity and the overall effect of AHI non-supine that favors the OAT group

We analyzed 208 patients from four included trials [25–27, 29] that reported this outcome. The analysis showed that the AHI non-supine was significantly lower in the OAT cohort than in the PT cohort (MD = 2.45 [1.06, 3.84], *P* = 0.0006). The pooled data showed homogeneity (*P* = 0.46, I^2^ = 0%) (Fig. 5).

#### Oxygen desaturation index (ODI), events/hour

**Fig. 6:**
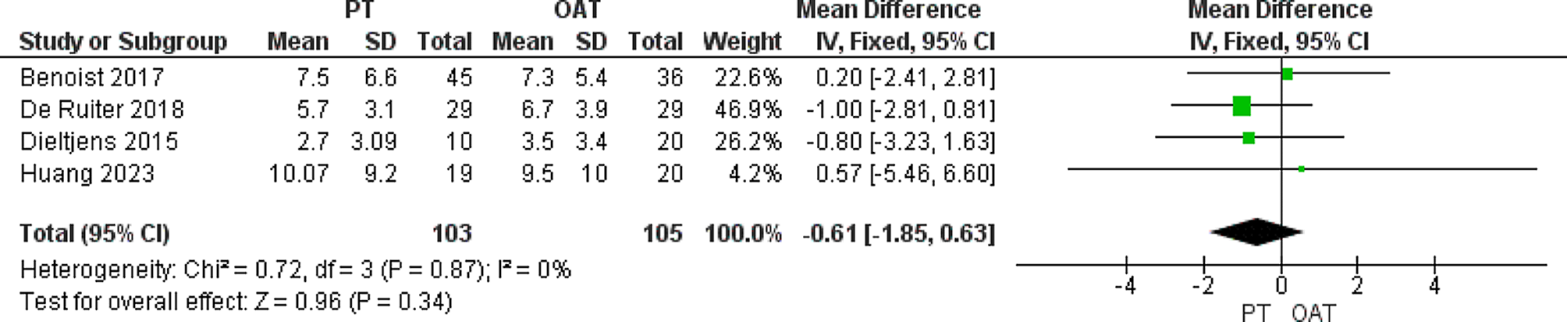
Heterogeneity and the overall effect of Oxygen desaturation index that does not favor any of both groups

We analyzed 208 patients from four included trials [25–27, 29] that reported this outcome. The analysis showed that both cohorts were similar without any substantial variations (MD= -0.61 [-1.85, 0.63], *P* = 0.34). The data was homogenous (*P* = 0.87, I^2^ = 0%) (Fig. 6).

#### Percentage supine sleep

**Fig. 7:**
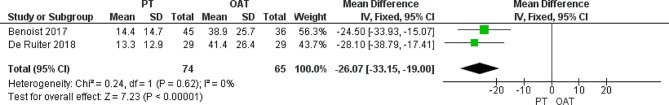
Heterogeneity and the overall effect of Percentage of supine sleep that favors the OAT group

After analyzing 139 participants from two included trials [25, 29] that reported the percentage of supine sleep. The analysis revealed that the PT cohort was associated with a significantly decreased percentage of supine sleep compared to the OAT cohort (MD= -26.07 [-33.15, -19.00], *P* = 0.0001). The data was homogenous (*P* = 0.62, I^2^ = 0%) (Fig. 7).

#### Sleep efficiency

**Fig. 8:**
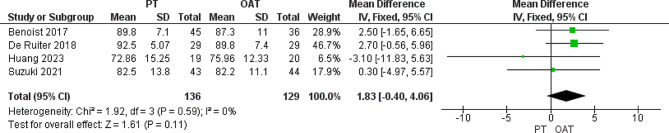
Heterogeneity and overall effect of sleep efficiency that does not favor any of both groups

Four studies [25, 27–29] reported the sleep efficiency of the participants. The sleep efficiency was the same in both cohorts without any significant variations (MD = 1.83 [-0.40, 4.06], *P* = 0.11). The data was homogenous (*P* = 0.59, I^2^ = 0%) (Fig. 8).

#### Arousal index (AI)

**Fig. 9:**
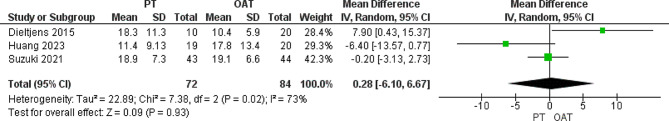
Heterogeneity and overall effect of the Arousal index that does not favor any of both groups

The AI outcome was reported by three studies [26–28]. Our analysis revealed that both cohorts had similar arousal index without substantial differences (MD = 0.28 [-6.10, 6.67], *P* = 0.93). The data was heterogeneous (*P* = 0.02, I^2^ = 73%) (Fig. 9).

#### Epworth sleepiness scale (ESS) score

**Fig. 10:**
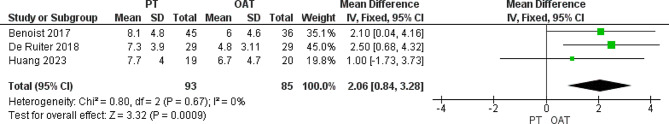
Heterogeneity and the overall effect of the Epworth Sleepiness Scale (ESS) score that favors the OAT group

Three studies [25, 27, 29] reported the ESS scores of the included participants. The ESS score was significantly lower with the OAT cohort than with the PT cohort (MD = 2.06 [0.84, 3.28], *P* = 0.0009). The data was homogenous (*P* = 0.67, I^2^ = 0%) (Fig. 10).

#### Functional outcomes of sleep questionnaire (FOSQ)

**Fig. 11:**
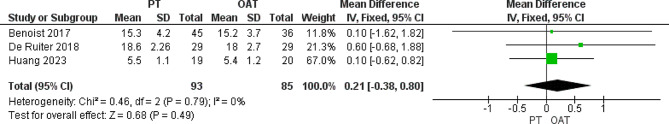
Heterogeneity and overall effect of Functional outcomes of sleep questionnaire (FOSQ) that does not favor any of both groups

Three studies [25, 27, 29] reported the FOSQ outcome. The analysis showed that there were no significant variations between both cohorts (MD = 0.21 [-0.38, 0.80], *P* = 0.49). The data was homogenous (*P* = 0.79, I^2^ = 0%) (Fig. 11).

#### Adherence (≥ 4 h/night, ≥ 5 days/week)

**Fig. 12:**
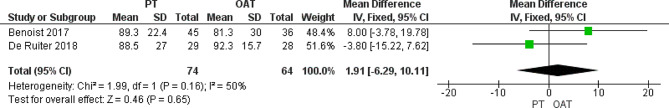
Heterogeneity and overall effect of Adherence that does not favor any of both groups

We analyzed the data of 138 participants from two included trials [25, 29]. The analysis showed that there were no significant variations between both cohorts (MD = 1.91 [-6.29, 10.11], *P* = 0.65). The data was homogenous (*P* = 0.16, I^2^ = 50%) (Fig. 12).

#### Mean SpO2

**Fig. 13:**
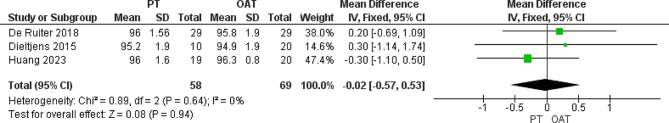
Heterogeneity and overall effect of Mean SpO2 that does not favor any of both groups

This outcome was reported by three studies [25–27]. The analysis showed that there were no significant variations between both cohorts (MD= -0.02 [-0.57, 0.53], *P* = 0.94). The data was homogenous (*P* = 0.64, I^2^ = 0%) (Fig. 13).

Our analysis and results rejected the null hypothesis as we found that Oral Appliance Therapy exhibited higher efficiency, leading to increased supine sleep percentage, more significant reductions in the Apnea-Hypopnea Index during non-supine positions, and lower scores on the Epworth Sleepiness Scale.

## Discussion

To our knowledge, this is the first study of its kind that compares the efficacy of PT and OAT for the management of individuals suffering from POSA. Our analysis showed that the AHI non-supine and the ESS scores were significantly lower in the OAT cohort than in the PT cohort (*P* = 0.0006) and (*P* = 0.0009), respectively. The PT cohort was associated with a significantly decreased percentage of supine sleep than the OAT cohort (*P* = 0.0001). There was no significant variation between the PT cohort and OAT cohort regarding total AHI (*P* = 0.15), AHI supine (*P* = 0.36), ODI (*P* = 0.34), sleep efficiency (*P* = 0.11), arousal index (*P* = 0.93), FOSQ (*P* = 0.49), Adherence (*P* = 0.65), and mean SpO2 (*P* = 0.94). The OAT cohort was associated with significantly lower AHI non-supine and ESS scores and an increased percentage of supine sleep than the PT cohort.

Unlike our results, Suzuki et al. [28] found that PT reduces respiratory events and supine sleep time and enhances the percentage of deep sleep more than OAT. Their study did have some drawbacks, though, since 45.0% and 46.2% of patients in the OAT and PT cohorts, respectively, did not respond to treatment adequately. This indicates that not all patients are candidates for these devices and that patient selection is crucial when using them. Marciuc et al. [30] conducted a systematic review and meta-analysis that evaluated the efficacy of oral appliances as a POSA treatment option. They concluded that OAT is an effective treatment for POSA as it improves breathing patterns by decreasing the AHI, which is consistent with our results.

Another systematic review and meta-analysis measured the impact of OAT on the quality of life of POSA patients. They found that OAT improves the quality of life of POSA patients [31]. Trindade et al. [32] included four studies with 83 adult patients and compared their results before and after OAT. They reported that OAT achieved a 79.5% reduction in AHI and a decrease in respiratory obstruction. Ravesloot et al. [33] performed a meta-analysis that assessed the efficacy of PT for managing POSA and involved six articles. Their analysis revealed that PT is an effective, simple, reversible, and cheap option for managing POSA. PT is an easy option for both patients and clinicians, and they reported that PT causes a reduction of the AHI.

The study conducted by Eijsvogel et al. provides evidence of the significance of compliance [34]. Although the therapeutic efficacy of PT and tennis ball technique (TBT) was equivalent, PT had better compliance. A mean disease alleviation of 48.6% for TBT and 70.5% for the new generation PT, respectively, was attained when compliance was taken into account [34]. Compliance issues are a problem with CPAP and, to a lesser extent, MAD treatment. A median usage of MAD therapy for 6.4 h per night was observed after 3 months, and a mean use of 6.1 h per night after one year was observed in two prospective small-scale studies with the advent of objective monitoring [35, 36]. Between 29% and 83% of CPAP users do not follow instructions. After just one night of use, 8–15% of patients decline CPAP therapy, and 20–40% stop using it after three months [37].

In 2023, ALQarni et al. [38] conducted a systematic review and meta-analysis that included eight cohort studies and ten clinical trials. These included studies compare different choices for managing POSA, such as PT, OAT, placebo, and CPAP. The primary conclusions of this systematic review and meta-analysis demonstrated that PT successfully lowered AHI and time spent in the supine position in individuals with POSA. The pooled data also showed a decrease in daytime sleepiness and a FOSQ, although these additional findings failed to reach a clinically significant difference. Additionally, the arousal index and sleep efficiency only slightly improved. When interpreting these findings, there are several things to take into account. The first is the variety of PT devices utilized and variations in the control groups of the included clinical trials. One study didn’t use any treatment. Two studies used CPAP, one study MAD, one study TBT, and two studies inactive PT treatment. The final two studies either employed combination therapy as a control or several comparators. As a result, pooling the results at follow-up was only achievable with PT when compared to baseline.

Benoist et al. [29] compare the efficacy of OAT and PT in treating individuals with mild to moderate positional POSA. They found that PT and OAT were similar in reducing ODI and AHI, which is consistent with our findings. In an analysis of 630 OSA/snoring individuals, Marklund et al. [39] found that with an odds ratio (OR) of 2.4, treatment success (AHI < 10) following oral appliance (OA) treatment could be predicted more accurately in women regardless of sleep position. Additionally, they stated that for men, the ORs for treatment success were 6.0 for POSA over non-POSA. Thirty-two patients (17 men and 15 females) with mild to moderate OSA were included in Makihara et al. [40] Every patient was assigned randomly to have a 75% mandibular advancement with an OA or a 50% mandibular advancement alone. They compared the AI, AHI, and ESS before and after treatment. The results showed that both groups’ AHI and AI greatly improved, with the group with 50% mandibular advancement showing the greatest improvement. For either group, there were no notable improvements in the ESS.

### Strengths

Our meta-analysis involved only RCTs with the exclusion of the observational studies. The analysis was double-arm analysis as all the included trials had two comparators that were the same (PT vs. OAT). We analyzed eleven outcomes that considered most of the outcomes that should be measured to assess the improvement of individuals suffering from POSA.

### Limitations

The main limitation of our meta-analysis is the small sample size, as so many people worldwide suffer from POSA. Additionally, the included trials had different follow-up periods; one had a 6-month follow-up period, and the other four had a 3-month follow-up period, which may affect our analysis. Also, not all of our outcomes were homogenous; some were heterogenous, and we could not solve this heterogeneity.

## Conclusion

The PT was comparable to OAT, and both were effective for managing OAs; however, OAT was more efficient and caused more reduction of AHI non-supine and ESS scores with an increase in the percentage of supine sleep than PT. Further research and more clinical trials should be conducted to get more evidence and measure both options’ effects after a long follow-up period.

### Electronic supplementary material

Below is the link to the electronic supplementary material.

Supplementary Material 1

## Data Availability

The datasets used and/or analysed during the current study available from the corresponding authors on reasonable request.
