# Supplementary material for: Oral appliance therapy vs. positional therapy for managing positional obstructive sleep apnea; a systematic review and meta-analysis of randomized control trials
Source: BMC Oral Health. 2024 Jun 7;24:666. doi: 10.1186/s12903-024-04277-8 (PMC11161918; doi:10.1186/s12903-024-04277-8)
Supplement: Supplementary file 1 — Supplementary Material 1 [file 12903_2024_4277_MOESM1_ESM.docx]

**In this study we searched the following databases; SCOPUS, PubMed, Cochrane Library, and Web of Science.**

**The summary of our search strategy and results of search.**

**SCOPUS:**

("Obstructive sleep apnea" OR "sleep apnea" OR "positional sleep apnea" OR "POSA") AND ("mandibular advancement" OR "oral appliance" OR "oral appliance devices" OR "OAT") AND ("positional therapy" OR "sleep position trainer")

Results: 482

**PubMed:**

("Obstructive sleep apnea" [MeSH Terms] OR "sleep apnea" OR "positional sleep apnea" OR "POSA") AND ("mandibular advancement" OR "oral appliance" OR "oral appliance devices" OR "OAT") AND ("positional therapy" OR "sleep position trainer")

Results: 379

**Cochrane Library:**

("Obstructive sleep apnea" OR "sleep apnea" OR "positional sleep apnea" OR "POSA") AND ("mandibular advancement" OR "oral appliance" OR "oral appliance devices" OR "OAT") AND ("positional therapy" OR "sleep position trainer")

**Results: 253**

**Web of Science:**

TS=("Obstructive sleep apnea" OR "sleep apnea" OR "positional sleep apnea" OR "POSA") AND TS=("mandibular advancement" OR "oral appliance" OR "oral appliance devices" OR "OAT") AND TS=("positional therapy" OR "sleep position trainer")

Results: 436
